# Obstetric complications and intelligence in patients on the schizophrenia-bipolar spectrum and healthy participants

**DOI:** 10.1017/S0033291719002046

**Published:** 2020-08

**Authors:** Laura Anne Wortinger, Kristine Engen, Claudia Barth, Vera Lonning, Kjetil Nordbø Jørgensen, Ole A. Andreassen, Unn Kristin Haukvik, Anja Vaskinn, Torill Ueland, Ingrid Agartz

**Affiliations:** 1 Department of Psychiatric Research, Diakonhjemmet Hospital, Oslo, Norway; 2 NORMENT, Division of Mental Health and Addiction, Oslo University Hospital & Institute of Clinical Medicine, University of Oslo, Oslo, Norway; 3 Department of Psychology, University of Oslo, Oslo, Norway; 4 Department of Clinical Neuroscience, Centre for Psychiatric Research, Karolinska Institute, Stockholm, Sweden

**Keywords:** Bipolar spectrum, cognition, intelligence, mood disorders bipolar, neuropsychology, obstetric complications, obstetric gynecology, premorbid IQ, schizophrenia, schizophrenia spectrum

## Abstract

**Background:**

Whether severe obstetric complications (OCs), which harm neural function in offspring, contribute to impaired cognition found in psychiatric disorders is currently unknown. Here, we sought to evaluate how a history of severe OCs is associated with cognitive functioning, indicated by Intelligence Quotient (IQ).

**Methods:**

We evaluated the associations of a history of OCs and IQ in 622 healthy controls (HC) and 870 patients on the schizophrenia (SCZ) – bipolar disorder (BIP) spectrum from the ongoing Thematically Organized Psychosis study cohort, Oslo, Norway. Participants underwent assessments using the NART (premorbid IQ) and the WASI (current IQ). Information about OCs was obtained from the Medical Birth Registry of Norway. Multiple linear regression models were used for analysis.

**Results:**

Severe OCs were equally common across groups. SCZ patients with OCs had lower performances on both premorbid and current IQ measures, compared to those without OCs. However, having experienced more than one co-occurring severe OC was associated with lower current IQ in all groups.

**Conclusions:**

Severe OCs were associated with lower IQ in the SCZ group and in the BIP and HC groups, but only if they had experienced more than one severe OC. Low IQ might be a neurodevelopmental marker for SCZ; wherein, severe OCs influence cognitive abilities and increase the risk of developing SCZ. Considering OCs as a variable of neurodevelopmental risk for severe mental illness may promote the development of neuroprotective interventions, improve outcome in vulnerable newborns and advance our ability to make clinical prognoses.

## Introduction

Exposure to a broad category of adversities during the fetal period or delivery, herein called obstetric complications (OCs), increases schizophrenia risk 1.5–5 fold (Cannon *et al*., 2002*b*; Geddes and Lawrie, 1995; Nosarti *et al*., 2012; Pugliese *et al*., 2019). Recent findings on the interaction of genetic and OCs risk factors have linked neural insult to schizophrenia (Ursini *et al*., 2018). The liability towards schizophrenia explained by genetic risk predicted the diagnosis in the presence of OCs, but not in their absence (Ursini *et al*., 2018). The influence of OCs in the risk for developing bipolar disorders is less clear (Nosarti *et al*., 2012; Pugliese *et al*., 2019; Scott *et al*., 2006).

Impaired cognition is common in psychiatric disorders (Van Rheenen *et al*., 2017), yet the degree of neurodevelopmental abnormalities is greater in schizophrenia than bipolar disorder (Demjaha *et al*., 2012), i.e. excessive neuromotor delays and cognitive difficulties were found in children who later developed schizophrenia compared to those who later developed affective disorders (Cannon *et al*., 2002*a*). Neuromotor abnormalities relate consistently to intelligence quotient (IQ) abilities and remain central in adult schizophrenia (Vaskinn *et al*., 2014). Even though schizophrenia and bipolar disorder are nosologically distinct, studies have revealed shared cognitive heterogeneity (Bora and Pantelis, 2015; Hill *et al*., 2013; Tamminga *et al*., 2014; Van Rheenen *et al*., 2016; Van Rheenen *et al*., 2017).

Certain features of cognition appear to be unaffected by disease processes (e.g. schizophrenia, dementia, depression), and cognitive measures such as word-reading ability can act as ‘hold tests’ to estimate premorbid IQ (Nelson and Willison, 1991). Other aspects of cognition seem to deteriorate with the course of illness; tests of more fluid intelligence provide an indication of current cognitive abilities in patients. In fact, lower premorbid IQ was related to more severe cognitive impairment across schizophrenia and bipolar diagnoses with larger deficits observed in schizophrenia (Van Rheenen *et al*., 2017). Premorbid IQ was highly predictive of psychotic disorders in an umbrella review (Khandaker *et al*., 2011; Radua *et al*., 2018; Woodberry *et al*., 2008), confirming the role of a cognitive antecedent in psychosis onset.

To date, little is known about the effects of having experienced OCs on cognition in severe mental illness. The most recent study of OCs’ association to cognitive functioning was a clinical cohort study of 157 patients in the year 2000 and showed no relationship within patients with affective psychosis and schizophrenia (Gilvarry *et al*., 2000). They did, however, find differences in premorbid IQ between patients and their first-degree relatives, where the greatest IQ disparity was found between patients with OCs and their relatives. The larger deficit in patients with OCs could not be explained by shared genetic factors alone and is an indication that OCs might contribute to the lower premorbid IQ in these patients.

The evolving inference is that the intra-uterine environment partly mediates the genomic risk of schizophrenia, which has basic implications for pathogenesis (Birnbaum and Weinberger, 2017; Ursini *et al*., 2018). Investigating the pre- and perinatal period can be challenging, as data are usually collected retrospectively. Small sample sizes, lack of statistical power to measure small-interactive effects and inefficient reporting on the pre- and perinatal period have restricted knowledge on the contribution of OCs in mental illness (Cannon *et al*., 2002*b*; Radua *et al*., 2018).

To overcome these limitations, we utilized the Medical Birth Registry of Norway (MBRN) to provide unique insight into pregnancy and birth information on 1492 patients and healthy controls in the Thematically Organized Psychosis (TOP) study cohort. The main goal of this study was to investigate putative effects of a history of OCs on adult cognition in schizophrenia and bipolar spectrum disorders. Neurodevelopmental implications of severe OCs might be marked cognitive impairments present before and after disease onset. A more severe disease progression indicated by a cognitive decline that reflects both early cognitive deficits and more severe course of pathology from the time of illness onset (Wells *et al*., 2015; Van Rheenen *et al*., 2017). Our study objectives in the clinical cohort of patients on the schizophrenia-bipolar spectrum and healthy controls aimed to: (1) investigate the prevalence of severe OCs across diagnoses through information obtained in the MBRN; (2) examine premorbid and current IQ of OCs-defined subgroups; (3) determine differential effects of OCs by diagnostic category; (4) assess the effects of the number of co-occurring OCs and (5) examine the degree to which OCs-defined subgroups could be differentiated on demographic and clinical measures.

## Materials and methods

### Participants

The TOP study is a thematic study research effort focused on the disease mechanisms of psychotic disorders and is the main study protocol at the Norwegian Centre for Mental Disorders Research (NORMENT, Oslo, Norway; www.med.uio.no/norment/english). Adult patients were recruited consecutively from psychiatric units (outpatient and inpatient) of public hospitals in the Oslo region, which have the complete catchment-based service area covering about one million inhabitants of Oslo's urban area. The hospitals are located in different parts of the city and are representative of the city's variation in sociodemographic characteristics. The healthy controls (HC) were randomly selected from the national population register and were residents in the same catchment area as the patients. After a complete description of the study, all participants gave written informed consent. The Regional Committee for Research Ethics and the Norwegian Data Inspectorate approved the study.

Exclusion criteria for both patients and HC were hospitalization for previous moderate or severe head injury, neurological disorder, medical conditions thought to interfere with brain function and age outside the range of 18–65 years. Additional exclusion criteria for HC were current or previous somatic illness and substance misuse disorders or dependency within the last 6 months. HC were also excluded if they or a first-degree relative had a lifetime history of severe psychiatric disorder.

All patients underwent thorough clinical investigation by trained psychologists and physicians. Clinical diagnoses were assessed using the Structured Clinical Interview for DSM-IV axis 1 disorder (SCID-I) module A-E (Spitzer *et al*., 1988). Psychosocial function was assessed with the Global Assessment of Function scale, split version (GAF; Pedersen *et al*., 2007). Current psychotic symptoms were rated by the use of the Positive and Negative Syndrome Scale (PANSS; Kay *et al*., 1987).

HC were interviewed by trained research assistants and examined with the Primary Care Evaluation of Mental Disorders (Prime-MD) to ensure no current or previous psychiatric disorders (Spitzer *et al*., 1994).

From the onset of the study in October 2002 until June 2015, the total subject sample (*n* = 1492) consisted of patients with a DSM-IV diagnosis within the *schizophrenia spectrum* (SCZ): schizophrenia (DSM-IV 295.1, 295.3, 295.6, and 295.9; *n* = 346), schizophreniform disorder (DSM-IV 295.4; *n* = 43), schizoaffective disorder (DSM-IV 295.7; *n* = 70) or psychosis not otherwise specified (DSM-IV 298.9; *n* = 148); or within the *bipolar spectrum* (BIP): Bipolar I disorder (DSM-IV 296.0–7; *n* = 164), Bipolar II disorder (DSM-IV 296.89; *n* = 80) or bipolar disorder not otherwise specified (DSM-IV 296.80; *n* = 19); and HC (*n* = 622).

### Obstetric complications

Birth data were collected from the national MBRN. In Norway, there is mandatory reporting on all births after gestational week 16 (online Supplementary material – Appendix 1: MBRN form from 1967–1998). MBRN data were scored for the presence and severity of OCs (McNeil and Sjöström, 1995) by two physicians (UKH and KE) who were blinded to patient/ control status (inter-rater reliability = 0.94).

The validated McNeil–Sjöström scale (McNeil *et al*., 1994; McNeil and Sjöström, 1995) includes several hundred events of potential harm to the fetus/offspring, each classified according to severity on an ordinal scale from 1 to 6. As in other reports (Nicodemus *et al*., 2008; Haukvik *et al*., 2014), an incidence of severe OCs was reported in those participants who had experienced one or more complications of a grade 5 or 6. Subjects with complications of grade 4 and below were classified as having an absence of severe OCs. A complication of grade 5 is defined as an event that is ‘potentially clearly greatly relevant/harmful’ to the central nervous system of the developing fetus/offspring, and a complication of grade 6 is defined as an event that causes ‘very great harm to or deviation in offspring’ (McNeil and Sjöström, 1995). Examples of grade 5 or 6 are as follows: severe preeclampsia, bleeding before 28 weeks, asphyxia, discolored placenta/amniotic fluid, emergency caesarean delivery, preterm birth ⩽35 weeks, low Apgar score (0–3 at 1 min or 0–7 at 5 min), bleeding during labor, low birth weight ⩽2000 g and eclampsia (see online Supplementary material – Note: Obstetric complications for more details).

### IQ measures

Current IQ was evaluated using the Wechsler Abbreviated Scale of Intelligence (WASI) full scale IQ assessment (Wechsler, 2007). The National Adult Reading Test (NART) was designed to give an indication of cognitive functioning preceding symptoms of disease and word-reading skills are significantly correlated with Wechsler-based IQ scores (Nelson and Willison, 1991). The reliability and validity of the Norwegian language version of the NART have been established (Sundet and Vaskinn, 2008). The premorbid IQ estimate was calculated using the WASI full scale IQ formula (Sundet and Vaskinn, 2008). Cognitive decline was calculated by subtracting premorbid IQ from current IQ scores (current IQ – premorbid IQ) and will be referred to as IQ difference scores.

### Statistical analyses

Statistical analyses were performed within the Statistical Package of Social Sciences (SPSS), version 25 (IBM, USA; www.spss.com).

Demographic and clinical characteristics were compared between groups using chi-squared tests for categorical variables and analysis of variance (ANOVA) or covariance (ANCOVA) for continuous variables. Age, years of education and sex distributions were identified as potential confounders on cognitive variables, and we adjusted accordingly in between-group analyses. Subjects with missing data on any particular variable were omitted from analyses involving that variable, but were included in analyses for which all required variables were present.

Three separate multiple linear regressions were calculated for premorbid IQ, current IQ or IQ difference scores (as dependent variables), respectively. Age, education, sex, OCs, HC *v*. BIP, HC *v.* SCZ, OC*BIP and OC*SCZ were entered as independent variables. Premorbid IQ was estimated from NART total errors, and covariates (age, education and sex) were adjusted for in the regression models.

To assess differences in the number of OCs frequency between groups, we preformed chi-squared tests. Further, to examine if cognitive abilities were directly related to the number of co-occurring OCs that were rated 5 and 6 in each group, we performed two additional multiple regression analysis entering IQ variables as dependent variables and age, education level, sex, HC *v.* SCZ, HC *v.* BIP, One OC, Two or more (2+) OCs as independent variables. In the second regression, age, education level, sex, One OC, Two OCs and Three or more (3+) OCs were compared in only HC *v.* SCZ because the BIP group had too few cases of 3 or more OCs.

## Results

### Demographic and clinical variables

There were significant differences in sex distribution and education levels between the three participant groups (Table 1). Age did not differ between patient groups, but it did differ between patient groups and HC. Between the patient groups, age at illness onset, illness duration, PANSS scores and GAF scores were significantly different. In the BIP group, age of illness onset was earlier than in the SCZ group. The duration of illness was longer in the BIP group than in the SCZ group. A greater degree of clinical symptoms was found in the SCZ group compared to the BIP group and functioning was significantly reduced in the SCZ group, as well. Age at illness onset, illness duration, PANSS and GAF variables did not differ as a function of OCs within patient groups. Current IQ and IQ difference scores differed between groups. Negative IQ difference scores indicated a discrepancy between current IQ and premorbid IQ. Premorbid IQ estimates did not differ between the BIP group and HC, but differed between the SCZ group and HC. Across all three groups, there were no significant differences in birth weight or gestational age.

**Table 1.** Demographic and clinical characteristics

	HC		BIP		SCZ		HC *v*. BIP		HC *v*. SCZ		BIP *v*. SCZ	
	*N* = 622		*N* = 263		*N* = 607		χ^2^/*t*	*p*	χ^2^/*t*	*p*	χ^2^/*t*	*p*
Sex (% female)	0.46		0.63		0.39		19.98	**0.000**	6.92	**0.009**	42.04	**0.000**
	Mean	s.d.	Mean	s.d.	Mean	s.d.						
Age (years)	30.06	6.91	27.56	6.34	27.04	6.50	−5.12	**0.000**	−7.96	**0.000**	−1.10	0.278
Education (years)	14.32	2.25	13.27	2.26	12.17	2.35	−6.00	**0.000**	−15.85	**0.000**	−6.10	**0.000**
Birth weight (grams)^a^	3521	538	3556	558	3481	576	1.40	0.162	−1.57	0.117	−2.38	0.018
Gestation age (weeks)	39.91	1.78	39.88	1.93	39.61	2.49	−0.14	0.887	−2.42	0.016	−1.57	0.117
Age at illness onset			19.03	5.99	21.89	6.17					6.28	**0.000**
Illness duration (years)			8.58	6.45	5.21	5.35					−7.92	**0.000**
PANSS			45.98	10.06	62.45	16.62					14.71	**0.000**
General			25.66	5.80	32.04	8.40					11.07	**0.000**
Negative			10.33	3.64	15.41	6.24					12.15	**0.000**
Positive			10.06	3.65	14.94	5.16					13.73	**0.000**
GAF-S			56.86	11.37	42.92	11.89					−15.96	**0.000**
GAF-F			55.19	12.95	44.58	12.21					−11.47	**0.000**
Premorbid IQ^b^	112.99	1.72	112.22	1.71	110.04	1.75	0.48	0.631	−4.79	**0.000**	−3.61	**0.000**
Current full scale IQ^c^	113.30	5.16	108.74	4.89	100.79	5.26	−2.19	**0.028**	−10.33	**0.000**	−5.32	**0.000**
IQ difference score^d^	0.34	3.64	−3.45	3.39	−8.45	3.72	−2.81	**0.005**	−9.19	**0.000**	−4.20	**0.000**

a Adjusted for sex; s.d. (standard deviation); HC (healthy controls); BIP (bipolar spectrum); SCZ (schizophrenia spectrum); PANSS (Positive and Negative Syndrome Scale); GAF-S (Global Assessment of Functioning- symptoms); GAF-F (Global Assessment of Functioning-functioning). Bold numbers represent significant values.

b NART premorbid IQ estimate adjusted for age, education and sex (HC: *N* = 582, BIP: *N* = 230 and SCZ: *N* = 411).

c WASI full scale IQ assessment adjusted for age, education and sex (HC: *N* = 580, BIP: *N* = 235 and SCZ: *N* = 428).

d IQ difference score (current IQ – premorbid IQ) adjusted for age, education and sex (HC: *N* = 579, BIP: *N* = 228 and SCZ: *N* = 396)

### Obstetric complications

There were no significant differences in the frequency of OCs between groups (Table 2). However, two of the complications (preterm and low birth weight) were more frequent in the group with SCZ patients, compared to the HC. Bleeding during labor occurred more often in the HC in comparison to the SCZ group. The most frequent OCs across groups were asphyxia (⩾50%) and discolored placenta/amniotic fluid (⩾39%).

**Table 2.** Frequency of obstetric complications

	HC		BIP		SCZ		HC *v*. BIP		HC *v*. SCZ		BIP *v*. SCZ	
	*N* = 622		*N* = 263		*N* = 607		χ^2^	*p*	χ^2^	*p*	χ^2^	*p*
	*N*	%	*N*	%	*N*	%						
Obstetric complications	166	0.27	85	0.32	170	0.28	2.89	0.089	0.27	0.604	1.65	0.199
Asphyxia	83	0.50	45	0.53	87	0.51	0.20	0.659	0.05	0.829	0.07	0.790
Discolored placenta/amniotic fluid	71	0.43	37	0.44	67	0.39	0.01	0.909	0.39	0.531	0.40	0.528
Emergency C-section	10	0.06	4	0.05	14	0.08	0.19	0.667	0.62	0.431	1.08	0.300
Preterm ⩽35 weeks	13	0.08	8	0.09	28	0.16	0.18	0.669	5.85	**0.016**	2.33	0.127
Low Apgar score	5	0.03	3	0.04	12	0.07	0.05	0.825	2.86	0.091	1.28	0.259
Bleeding during labor	33	0.20	12	0.14	19	0.11	1.27	0.260	4.86	**0.027**	0.46	0.498
Bleeding before 28 weeks	8	0.05	3	0.04	16	0.09	0.22	0.637	2.67	0.102	2.84	0.092
Low birth weight ⩽2000 g	4	0.02	4	0.05	15	0.09	0.96	0.327	6.48	**0.011**	1.39	0.238
Other birth complications	11	0.07	7	0.08	21	0.12	0.22	0.640	3.20	0.074	0.98	0.321

HC, healthy controls; BIP, bipolar spectrum; SCZ, schizophrenia spectrum. Bold numbers represent significant values.

### IQ measures

Multiple linear regression analyses revealed that the independent variables predicted premorbid IQ, current IQ and IQ difference scores in the overall models (Table 3). Each model explained 24%, 31% and 23% of the variance in the change of IQ scores, respectively. We found an OCs by SCZ group interaction on premorbid and current IQ variables. An interaction effect indicated that within the SCZ group, patients with a history of OCs had lower premorbid and current IQ compared to SCZ patients without a history of OCs. This was not the case for the HC or BIP groups.

**Table 3.** Multiple regression analyses by premorbid IQ, current IQ and IQ difference scores

Level of IQ performance	*B*	*β*	*t*	*p*	*F*	df	*p*	adj. *R*^2^
*Premorbid IQ*^a^								
Overall model					48.66	8, 1222	**0.000**	0.24
(Constant)	101.34		129.26	0.000				
Age	0.71	0.109	3.88	**0.000**				
Education level	0.65	0.361	12.29	**0.000**				
Sex	0.24	0.027	1.07	0.284				
Severe OCs	0.21	0.021	0.58	0.561				
HC *v*. BIP	0.32	0.028	0.85	0.395				
HC *v*. SCZ	−0.97	−0.103	−3.13	**0.002**				
OC*BIP	−0.57	−0.030	−0.86	0.391				
OC*SCZ	−1.36	−0.084	−2.36	**0.018**				
*Current IQ*^b^								
Overall model					71.83	8, 1242	**0.000**	0.31
(Constant)	82.63		36.67	0.000				
Age	−0.13	−0.065	−2.44	**0.015**				
Education level	2.28	0.418	15.00	**0.000**				
Sex	3.19	0.118	4.94	**0.000**				
Severe OCs	0.27	0.009	0.26	0.796				
HC *v*. BIP	−1.50	−0.043	−1.41	0.160				
HC *v*. SCZ	−7.17	−0.252	−8.04	**0.000**				
OC*BIP	−1.52	−0.027	−0.80	0.422				
OC*SCZ	−3.73	−0.078	−2.28	**0.023**				
*IQ difference score*^c^								
Overall model					46.67	8, 1202	**0.000**	0.23
(Constant)	−17.92		−9.23	0.000				
Age	−0.21	−0.128	−4.52	**0.000**				
Education level	1.59	0.359	12.07	**0.000**				
Sex	3.06	0.140	5.46	**0.000**				
Severe OCs	0.12	0.005	0.13	0.893				
HC *v*. BIP	−1.86	−0.067	−2.03	**0.042**				
HC *v*. SCZ	−5.69	−0.244	−7.36	**0.000**				
OC*BIP	−1.00	−0.022	−0.61	0.540				
OC*SCZ	−2.34	−0.058	−1.63	0.104				

HC, healthy controls; BIP, bipolar spectrum; SCZ, schizophrenia spectrum; df, degrees of freedom. Bold numbers represent significant values.

a NART premorbid IQ estimate adjusted for age, education and sex (HC: *N* = 582, BIP: *N* = 230 and SCZ: *N* = 411).

b WASI full scale IQ assessment adjusted for age, education and sex (HC: *N* = 580, BIP: *N* = 235 and SCZ: *N* = 428).

c IQ difference score (current IQ – premorbid IQ) adjusted for age, education and sex (HC: *N* = 579, BIP: *N* = 228 and SCZ: *N* = 396).

Because OCs are not isolated events and commonly occur together (McNeil and Sjöström, 1995), we assessed the number of OCs that were rated 5 and 6 in each group using chi-squared analyses. We found no statistically significant differences (χ^2^ (2) = 1.63, *p* = 0.442) in the number of co-occurring OCs (1 and 2+) between the SCZ group (1 OC = 56%; 2 + OCs = 44%), the BIP group (1 OC = 61%; 2 + OCs = 39%) and HC (1 OC = 63%; 2 + OCs = 37%). In the BIP group, there were only 3 cases with 3 + OCs, so the BIP group was not included in these analyses. We found no statistically significant differences (χ^2^ (2) = 4.87, *p* = 0.088) in the number of co-occurring OCs (1, 2 and 3+) between the SCZ group (1 OC = 56%; 2 OCs = 31%; 3 + OCs = 13%) and HC (1 OC = 63%; 2 OCs = 31%; 3 + OCs = 6%).

**Fig. 1.**
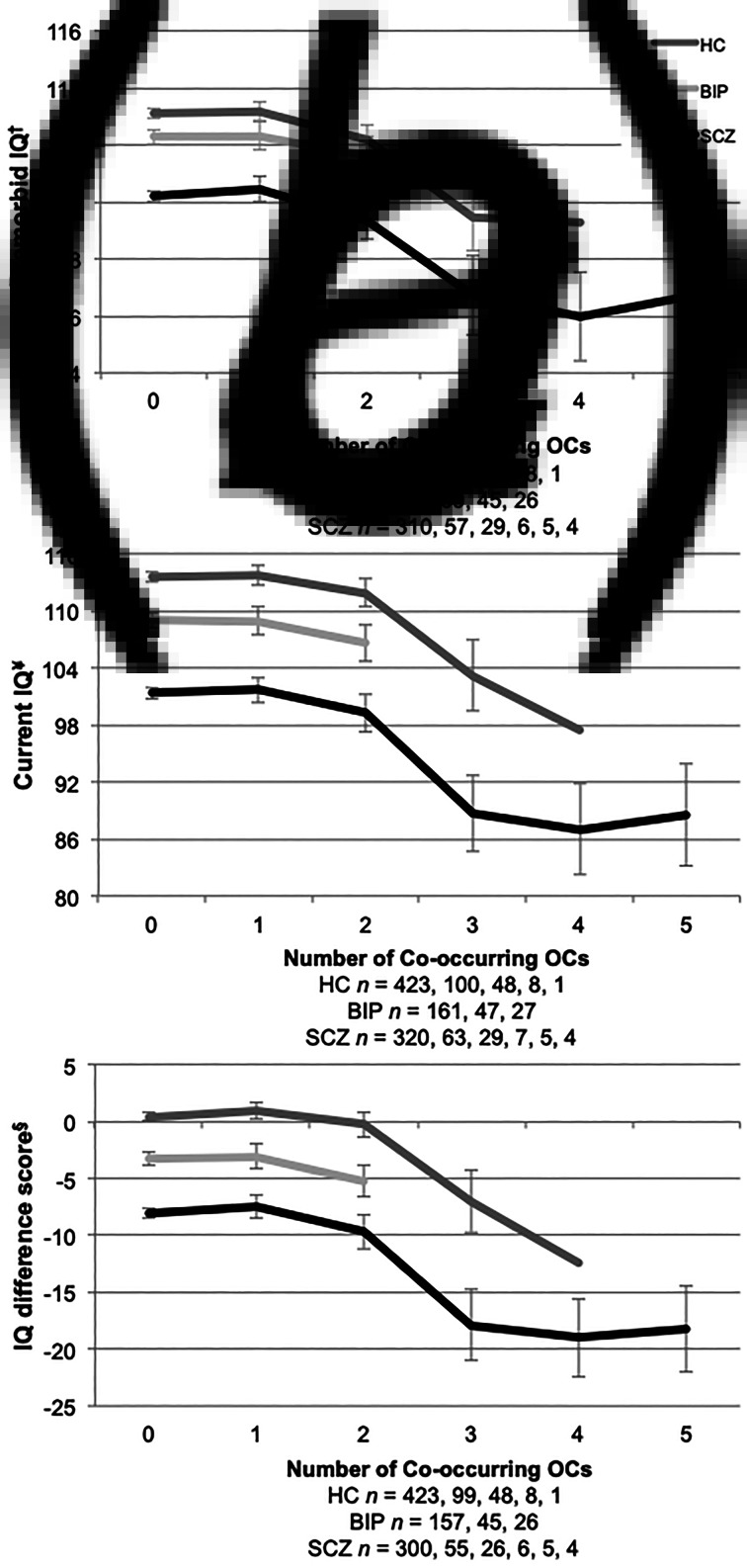
IQ across the number of co-occurring severe OCs. HC, healthy controls; BIP, bipolar spectrum; SCZ, schizophrenia spectrum. (*a*) ^†^NART premorbid IQ estimate; (*b*) ^¥^WASI full scale assessment; and (*c*) ^§^IQ difference score (current IQ – premorbid IQ). The numbers below the *x-axis* refer to the number of co-occurring OCs in participant histories. Error bars represent ±2 standard errors.

Furthermore, we found premorbid IQ scores were significantly lower with more than 2 severe OCs in both adult patients with SCZ and HC (online Supplemental materials: Table S1B and Fig. 1*a*). This was not the case for BIP group, as they did not differ from the HC on premorbid IQ measures. Current IQ measures were significantly lower if more than one severe OC was experienced, and this effect was observed in all groups (online Supplemental materials: Table S1A and Fig. 1*b*). IQ difference scores (current IQ – premorbid IQ) showed a significant decrease in IQ scores in those that had experienced more than one severe OC, also seen in all groups (online Supplemental materials: Table S1A and Fig. 1*c*). In those with 2 + OCs, asphyxia-related OCs were identified in 88% (50 out of 57) of the healthy controls, 81% (21 out of 26) of patients in the bipolar group and 71% (29 out of the 41) of patients in the schizophrenia group. We identified 15 schizophrenia patients with current IQ below 70 in our sample, and 33% (5 out of 15) had severe OCs in their histories.

## Discussion

We found severe OCs to be equally common across groups, and the number of OCs co-occurring was also similar. An interaction effect indicated that within the schizophrenia group, patients with a history of OCs had lower premorbid and current IQ compared to schizophrenia patients without a history of OCs. To have experienced more than two co-occurring OCs was specifically associated with lower premorbid IQ in both patients within the schizophrenia group and healthy controls, but not in the bipolar group. However, more than one co-occurring OC was related to lower current IQ and greater decreases in IQ difference scores in all participant groups.

Despite scientific reports that have stressed the role of OCs as a risk factor for the development of schizophrenia (Cannon *et al*., 2002*b*; Geddes and Lawrie, 1995; Nosarti *et al*., 2012; Pugliese *et al*., 2019), the precise nature of their relationship to cognitive functioning in psychiatric disorders is unknown. We report that severe OCs occurred in 28% of patients within the schizophrenia group, but we also observed similar numbers in both the bipolar patient group and healthy control group, which is comparable to what has been reported in other studies (Haukvik *et al*., 2009; Nicodemus *et al*., 2008; Ursini *et al*., 2018). These findings support the idea that having a history of OCs might be an additive/interactive risk for the development of a psychiatric disorder (Walter and Holford, 1978). Since OCs were equally occurring in all groups, psychiatric disorders must be mainly determined by other factors and not directly caused by OCs. Severe OCs might be an additional factor, wherein their presence further increases the probability of developing a condition with a more severe cognitive course. In this study, we found that the associations between OCs and IQ were stronger in patients within the schizophrenia group, compared to the bipolar group, but were also observed in the healthy controls.

IQ is the most discriminating neuropsychological variable between patients with schizophrenia and affective disorders (Goldberg *et al*., 1993). We found that patients with schizophrenia and OCs had lower premorbid and current IQ than patients who had not experienced OCs. Severe OCs in this patient group can be presumed to have early neurodevelopmental consequences on cognition, as reduced IQ was present before illness onset. Common genetic variants associated with schizophrenia are enriched for associations with lower intelligence (Smeland *et al*., 2019) and intracranial volume (Smeland *et al*., 2018), a proposed biomarker of brain development (Woodward and Heckers, 2015). Lower IQ performance might indicate brain alterations and differing developmental trajectories within schizophrenia. Even though we did not identify differences on clinical measures between OCs-related subgroups, more studies are need to clarify the relationship between OCs, genetic risk, brain changes and clinical characteristics.

We found that having more than one severe OC, which is clearly harmful to the developing central nervous system of the fetus/offspring (McNeil and Sjöström, 1995), was specifically associated to lower current IQ and greater IQ difference scores (current IQ – premorbid IQ) in both patient groups and healthy controls, which indicates a decline in cognition. The significant discrepancies between current and premorbid IQ in both patient groups might imply that common pathogenic processes operate once illness has begun. Additionally, processes may have started earlier in schizophrenia, as IQ did not differ between the bipolar group and healthy controls, premorbidly, which gives an indication that there might be better resilience in bipolar disorder. Yet, lower current IQ and greater IQ difference scores in those who had experienced more than one OC might suggest a more vulnerable neurodevelopmental subgroup of both patient groups and healthy controls who are less resilient to age-related processes or pathological processes beginning later in life.

We found that 50% of all severe OCs were related to asphyxia at birth (i.e. deprivation of oxygen). Of the participants with more than one OC, we identified asphyxia-related OCs in 88% of healthy controls, 81% of patients in the bipolar group and in 71% of patients in the schizophrenia group. We observed a pattern of lower IQ as the number of OCs increased that might reflect the magnitude of neurodevelopmental insult of OCs on cognitive brain development. Asphyxia might specifically impact the developing brain. It appears that both a greater number of harmful OCs and asphyxia experienced at birth influence adult IQ, more so in vulnerable individuals who later develop a severe mental illness, but a series of OCs do not necessarily lead to a disorder. A direction for future research might be to improve knowledge of neural injury due to OCs and implement neuroprotective interventions for better outcome in vulnerable newborns.

Current pharmacological treatment for schizophrenia mainly improves positive symptoms, but has no documented improvement on cognitive impairments, which together with negative symptoms are the most disabling features of schizophrenia long-term (Fusar-Poli *et al*., 2015). We identified 15 schizophrenia patients with current IQ below 70 in our sample. Excluding this group from analyses did not alter group proportions, means or results, and doing so, would not reflect the number of patients with compromised intellectual function in the schizophrenia population (Wells *et al*., 2015). Severe OCs were found in 33% of these patients, which might have been an additive risk factor for schizophrenia. We realize that there are other non-purely genetic risk factors, defined as socio-demographic, parental, later factors and antecedents (Radua *et al*., 2018), that may increase the risk or decrease any protective effects and influence the likelihood of developing a psychiatric disorder. Urbanization, migration, childhood social withdrawal, childhood trauma, Toxoplasma gondii Immunoglobulin G, and non-right handedness might interact or be additive risks (Radua *et al*., 2018) to a genetic predisposition that operates by impairing the resilience of the fetal and neonatal brain (Murray *et al*., 2017). The neurodevelopmental significance of having experienced severe OCs is that cognitive deficits are major aspects of treatment-resistant schizophrenia. Therefore, we need to explore other avenues of treatment and optimize perinatal care in vulnerable individuals, for instance in mothers with a family history of severe psychosis.

Strengths of this study were the use of the Medical Birth Registry of Norway data, which allows for a more accurate and prospective assessment of OCs in severe mental illness. Our study provides *a priori* knowledge about the pre- and perinatal environment (indicated with OCs) in patients who later develop psychiatric disorders. We observed normal-ranged IQ means (i.e. ≈100) across groups, yet we were still able to identify adverse effects of OCs on IQ measures. Importantly, it reveals clinical evidence on early life factors and their possible impact on cognition in adult patients, which is currently missing in the field (Radua *et al*., 2018). Including OCs as a component of risk may advance our ability to make prognoses in psychiatric disorders, which might soon compliment genetic vulnerability or other predictors.

The use of the NART as an index of prior intellectual ability (rather than current) is well established with high retrospective validity. In fact, a 66-year follow up study in healthy individuals found that NART performance at age 77 and IQ at age 11 had a highly significant correlation (*r* = 0.73, *p* < 0.001) (Crawford *et al*., 2001). The validation for the Norwegian version of the NART for patients with schizophrenia and bipolar disorders also revealed highly significant correlations between estimated and actual IQ (*r* = 0.64, *p* < 0.001; *r* = 0.49, *p* < 0.001, respectively) (Sundet and Vaskinn, 2008). The use of the NART as an estimate of premorbid intellectual ability in clinical populations is supported by this series of results and is in line with the findings of the present study.

This study has some limitations. Exclusion criteria in the TOP study precluded recruitment of healthy controls if they had a first-degree relative with a lifetime history of severe psychiatric disorder. Further, despite a random selection from the population, it seems that the controls were performing above average on cognitive tests. A recent study indicates that large proportions of the genomic risk underlying schizophrenia also influence intelligence (Smeland *et al*., 2019). It is possible that even more pronounced effect of OCs on IQ would have been evident had more genetically varied controls been included in the analyses.

Although the NART is a validated measure of premorbid IQ (Sundet and Vaskinn, 2008), an actual IQ assessment administered before the onset of symptoms may more accurately describe participants, especially in cases of extreme scores (Mathias *et al*., 2007).

Even though we did not find an association between OCs and premorbid IQ in the bipolar group, this group had half the sample size of the other two groups in our study. Since each type of OC event is infrequent in a population, the smaller sample size in this group might lack the statistical power to measure small and interactive effects. Cognitive decline, indicated by IQ difference scores, in the bipolar patients with OCs was significantly lower when the number of co-occurring OCs was taken into account.

In conclusion, the relevance of this study is that a history of severe OCs seems to be associated with cognitive functioning in adult participants. These findings add to new reports indicating that genetic risk for schizophrenia is found to start in the womb, interacting with placental integrity (Ursini *et al*., 2018). Even though the placenta might be a key environmental factor for psychopathology, placenta status is also of major importance for IQ (Jacobs *et al*., 2001; Patterson, 2007). Low IQ might be a marker for schizophrenia development and an experience of OCs appears to increase the risk. A better understanding of early pre- or perinatal factors such as OCs and genetic risk in the context of brain development could advance the role of perinatal care for reducing the burden of severe mental illness. Also, it may improve strategies for primary prevention of schizophrenia.

## Supplementary material

For supplementary material accompanying this paper visit https://doi.org/10.1017/S0033291719002046. click here to view supplementary material
