# Supplementary material for: Obstetric complications and intelligence in patients on the schizophrenia-bipolar spectrum and healthy participants
Source: Psychol Med. 2019 Aug 28;50(11):1914–22. doi: 10.1017/S0033291719002046 (PMC7477368; doi:10.1017/S0033291719002046)
Supplement: Supplementary file 1 [file S0033291719002046sup001.docx]

# Obstetric complications and intelligence in patients on the schizophrenia-bipolar spectrum and healthy participants

Laura Anne Wortinger PhD^1, 2^^[[1]](#footnote-1)^, Kristine Engen MD^1, 2^, Claudia Barth PhD^1, 2^, Vera Lonning MD^1, 2^, Kjetil Nordbø Jørgensen PhD^1, 2^, Ole A. Andreassen MD PhD^2^, Unn Kristin Haukvik MD PhD^2^, Anja Vaskinn PhD^2^, Torill Ueland PhD^2, 4^ and Ingrid Agartz MD PhD^1,^ ^2, 4^

^1^Department of Psychiatric Research, Diakonhjemmet Hospital, Oslo, Norway

^2^NORMENT: Norwegian Centre for Mental Disorders Research, Institute of Clinical Medicine, University of Oslo, Oslo, Norway; Division of Mental Health and Addiction, Oslo University Hospital, Oslo, Norway

^3^Centre for Psychiatric Research, Department of Clinical Neuroscience, Karolinska Institute, Stockholm, Sweden

^4^Department of Psychology, University of Oslo, Oslo, Norway

## Supplementary material:

Table 1: Greater number of severe OCs predicts lower adult IQ

| **A** | Level of IQ performance | B | *β* | *t* | *p* |  | **B** | Level of IQ performance | B | *β* | *t* | *p* |
| --- | --- | --- | --- | --- | --- | --- | --- | --- | --- | --- | --- | --- |
|  | **Premorbid IQ^†^** |  |  |  |  |  |  | **Premorbid IQ^†^** |  |  |  |  |
|  | (Constant) | 101.57 |  | 129.55 | .000 |  |  | (Constant) | 101.43 |  | 116.92 | .000 |
|  | **Age** | 0.07 | .104 | 3.71 | **.000** |  |  | **Age** | 0.07 | .108 | 3.51 | **.000** |
|  | **Education level** | 0.65 | .361 | 12.25 | **.000** |  |  | **Education level** | 0.66 | .363 | 11.14 | **.000** |
|  | Sex | 0.25 | .029 | 1.12 | .261 |  |  | Sex | 0.17 | .019 | 0.69 | .489 |
|  | **HC vs. SCZ** | -1.33 | -.141 | -4.85 | **.000** |  |  | **HC vs. SCZ** | -1.24 | -.135 | -4.45 | **.000** |
|  | HC vs. BIP | 0.15 | .013 | 0.49 | .626 |  |  | One OC | 0.05 | .004 | 0.16 | .877 |
|  | One OC | -0.15 | -.013 | -0.51 | .613 |  |  | Two OCs | -0.27 | -.016 | -0.57 | .567 |
|  | 2+ OCs | -0.58 | -.040 | -1.56 | .120 |  |  | **3+ OCs** | -2.89 | -.100 | -3.61 | **.000** |
|  | Overall model |  |  |  |  |  |  | Overall model |  |  |  |  |
| ***F*** | 54.74 |  |  |  |  |  | ***F*** | 50.87 |  |  |  |  |
| ***df*** | 7, 1222 |  |  |  |  |  | ***df*** | 7, 992 |  |  |  |  |
| ***p*** | **.000** |  |  |  |  |  | ***p*** | **.000** |  |  |  |  |
| **R^2^** | .24 |  |  |  |  |  | **R^2^** | .26 |  |  |  |  |
|  |  |  |  |  |  |  |  |  |  |  |  |  |
|  | **Current IQ¥** |  |  |  |  |  |  | **Current IQ¥** |  |  |  |  |
|  | (Constant) | 83.41 |  | 37.06 | .000 |  |  | (Constant) | 81.91 |  | 33.23 | .000 |
|  | **Age** | -0.14 | -.071 | -2.66 | **.008** |  |  | **Age** | -0.13 | -.064 | -2.25 | **.025** |
|  | **Education level** | 2.28 | .417 | 14.97 | **.000** |  |  | **Education level** | 2.34 | .425 | 14.00 | **.000** |
|  | **Sex** | 3.22 | .119 | 4.98 | **.000** |  |  | **Sex** | 3.51 | .125 | 4.94 | **.000** |
|  | **HC vs. SCZ** | -8.16 | -.286 | -10.42 | **.000** |  |  | **HC vs. SCZ** | -7.77 | -.276 | -9.78 | **.000** |
|  | **HC vs. BIP** | -1.943 | -.056 | -2.179 | **.030** |  |  | One OC | -0.08 | -.002 | -0.08 | .933 |
|  | One OC | -0.54 | -.015 | -0.62 | .534 |  |  | Two OCs | -0.54 | -.010 | -0.40 | .689 |
|  | **2+ OCs** | -2.47 | -.056 | -2.31 | **.021** |  |  | **3+ OCs** | -11.13 | -.124 | -4.87 | **.000** |
|  | Overall model |  |  |  |  |  |  | Overall model |  |  |  |  |
| ***F*** | 81.55 |  |  |  |  |  | ***F*** | 81.68 |  |  |  |  |
| ***df*** | 7, 1242 |  |  |  |  |  | ***df*** | 7, 1007 |  |  |  |  |
| ***p*** | **.000** |  |  |  |  |  | ***p*** | **.000** |  |  |  |  |
| **R^2^** | 0.31 |  |  |  |  |  | **R^2^** | .36 |  |  |  |  |
|  |  |  |  |  |  |  |  |  |  |  |  |  |
|  | **IQ difference score^§^** |  |  |  |  |  |  | **IQ difference score^§^** |  |  |  |  |
|  | (Constant) | -17.26 |  | -8.91 | .000 |  |  | (Constant) | -18.57 |  | -8.77 | .000 |
|  | **Age** | -0.22 | -.135 | -4.76 | **.000** |  |  | **Age** | -0.21 | -.127 | -4.14 | **.000** |
|  | **Education level** | 1.58 | .358 | 12.03 | **.000** |  |  | **Education level** | 1.63 | .367 | 11.29 | **.000** |
|  | **Sex** | 3.07 | .140 | 5.49 | **.000** |  |  | **Sex** | 3.44 | .153 | 5.62 | **.000** |
|  | **HC vs. SCZ** | -6.30 | -.271 | -9.28 | **.000** |  |  | **HC vs. SCZ** | -5.99 | -.263 | -8.72 | **.000** |
|  | **HC vs. BIP** | -2.17 | -.078 | -2.83 | **.005** |  |  | One OC | 0.31 | .010 | 0.37 | .715 |
|  | One OC | 0.02 | .001 | 0.02 | .982 |  |  | Two OCs | -0.34 | -.008 | -0.29 | .774 |
|  | **2+ OCs** | -2.20 | -.061 | -2.38 | **.018** |  |  | **3+ OCs** | -9.07 | -.126 | -4.58 | **.000** |
|  | Overall model |  |  |  |  |  |  | Overall model |  |  |  |  |
| ***F*** | 53.66 |  |  |  |  |  | ***F*** | 54.88 |  |  |  |  |
| ***df*** | 7, 1202 |  |  |  |  |  | ***df*** | 7, 974 |  |  |  |  |
| ***p*** | **.000** |  |  |  |  |  | ***p*** | **.000** |  |  |  |  |
| **R^2^** | .24 |  |  |  |  |  | **R^2^** | .28 |  |  |  |  |

HC (healthy controls); BIP (bipolar spectrum); SCZ (schizophrenia spectrum); degrees of freedom (df); A IQ performances with 2+ OCs or B 3+ OCs. ^†^NART premorbid IQ estimate (HC -N: 0 OC = 425, 1 OC = 99, 2+ OCs = 58; BIP -N: 0 OC = 159, 1 OC = 45, 2+ OCs = 26; and SCZ -N: 0 OC = 310, 1 OC = 57, 2+ OCs = 44); ^¥^WASI full scale IQ assessment (HC -N: 0 OC = 423, 1 OC = 100, 2+ OCs = 57; BIP -N: 0 OC = 161, 1 OC = 47, 2+ OCs = 27; and SCZ -N: 0 OC = 320, 1 OC = 63, 2+ OCs = 45); ^§^IQ difference score (current IQ – premorbid IQ) (HC -N: 0 OC = 423, 1 OC = 99, 2+ OCs = 57; BIP -N: 0 OC = 157, 1 OC = 45, 2+ OCs = 26; and SCZ -N: 0 OC = 300, 1 OC = 55, 2+ OCs = 41).

### Note: Obstetric complications

Certain complications were categorized as a grade 5 or 6 due to the obvious severity of the condition in and of itself (e.g. maternal cancer such as leukemia was scored as a grade 5 complication and the presence of a tetanus uterus was scored as a grade 6). Other conditions required additional information that was sufficiently included in the Medical birth registry of Norway (MBRN). For example, a caesarean delivery can be scored as a grade 4 or 5, depending on if it was an elective procedure or an emergency. Other conditions, however, required further information that was not provided in the MBRN. As in the use of forceps during delivery, forceps can be scored as a grade 3, 4, 5 or 6, depending on its use in relation to the location of the fetal head, but the MBRN does not provide fetal head location. In such cases, the McNeil–Sjöström scale recommends that the condition be scored as a grade-4 complication, in lieu of additional information.

The MBRN information has specific limitations, but the frequency of OCs in this study may be related to the information available about its presence or absence. When applying the McNeil-Sjöström scale directly to medical records, it is often more challenging to rule out at least one serious OCs than confirm its presence. For example, if the medical record indicated the use of forceps during delivery, the MBRN does not include additional information on its use in relation to the fetal head, so we had to exclude the participant from being categorized as having experienced a severe OC. However, we were able to include the participant when other clear OCs (e.g. preeclampsia, asphyxia) were reported. The information provided in the MBRN was restrictive in certain conditions, but these limitations were analogues across participant groups.

### Appendix 1: MBRN form from 1967-1998

##

1. Author for correspondence: Dr. Laura Wortinger, E-mail: [l.a.w.bakke@medisin.uio.no](mailto:l.a.w.bakke@medisin.uio.no) [↑](#footnote-ref-1)
